# Peptides containing SARS-CoV-2 spike-protein residues are antagonists of α7 and α9α10 nAChRs and modulate interleukin-1β release from human monocytic THP-1 cells

**DOI:** 10.1016/j.jbc.2026.113391

**Published:** 2026-08-05

**Authors:** Arik J. Hone, Katrin Richter, Zoha Riaz, Joseph Wakkuri, Bassel Tekarli, Joanna Gajewiak, Veronika Grau, J. Michael McIntosh

**Affiliations:** 1 School of Biological Sciences, University of Utah, Salt Lake City, Utah, USA; 2 MIRECC, George E. Wahlen Veterans Affairs Medical Center, Salt Lake City, Utah, USA; 3 Laboratory of Experimental Surgery, Department of General and Thoracic Surgery, German Center for Lung Research (DZL), Cardiopulmonary Institute (CPI), Justus-Liebig University Giessen, Giessen, Germany; 4 Department of Natural Sciences, Bonn-Rhein-Sieg University of Applied Sciences, Rheinbach, Germany; 5 Department of Psychiatry, University of Utah, Salt Lake City, Utah, USA; 6 George E. Wahlen Veterans Affairs Medical Center, Salt Lake City, Utah, USA

**Keywords:** SARS-CoV-2, COVID-19, spike protein, furin cleavage site, cytokine storm, nicotinic acetylcholine receptors, α-conopeptides

## Abstract

Immune system dysregulation during COVID-19 illness remains an area of intense investigation. Excessive release of proinflammatory cytokines and multi-organ injury suggest aberrant control of the immune system. Given the established role of nicotinic acetylcholine receptors (nAChRs) in modulating inflammatory responses, we explored the possibility that the SARS-CoV-2 spike-protein may directly perturb this regulatory pathway. The spike protein contains a furin cleavage-site with sequence similarities to α-neurotoxins known to antagonize α7, α9, and α10 nAChR subtypes. To test whether this region of the spike protein can influence nAChR function, we synthesized short peptides corresponding to putative nAChR-interacting domains from several SARS-CoV-2 variants. Electrophysiological recordings from *Xenopus laevis* oocytes expressing human nAChRs revealed potent, virus-variant dependent inhibition of nAChR activity. In human monocytic THP-1 cells, these peptides reversed acetylcholine-mediated suppression of interleukin-1β release. Our findings identify a potential molecular mechanism by which SARS-CoV-2 may directly suppress nAChR signaling on immune cells thereby amplifying inflammatory cytokine release. This work supports the broader concept that structural motifs present in the SARS-CoV-2 spike-protein can disrupt cholinergic anti-inflammatory pathways and elucidates a mechanism that may contribute to the pathophysiology and immune system dysregulation that can occur in severe COVID-19.

The emergence of severe acute respiratory syndrome-coronavirus-2 (SARS-CoV-2) late in 2019 furthered interest in how zoonotic viruses infect human hosts (1). With respect to COVID-19, it has been of particular interest how the bat SARS-CoV-like virus, RaTG13, became capable of infecting humans and other mammals since this virus has, until relatively recently, only been found in members of the Rhinolophidae bat family (2, 3, 4). Evidence suggests that SARS-CoV evolved from a progenitor by, in part, mutation of the spike protein, which enabled infection of other mammals. For example, phylogenetic analysis of viral origin suggested that one of the first known mammalian non-Chiroptera species to become infected by RaTG13 was the racoon dog (*Nyctereutes procyonoides*) (4, 5) which is native to Southeast China where SARS-CoV-1 is believed to have originated (6). Subsequent zoonotic virus transfer events have been postulated to have occurred between raccoon dogs and Maylan pangolins (*Manis javanica*) and thereafter from pangolins to humans, spawning the COVID-19 pandemic (7, 8).

One of the distinctions between SARS-CoV-2 and other β-coronaviruses is the presence of a four amino-acid insert (^682^RRAR^685^) in the spike protein that forms a potential cleavage site (^682^RRAR↓S^686^) for the enzyme furin (9, 10). Neither RaTG13 nor the SARS-CoV-1 strain isolated from pangolins contain this furin cleavage-site (FCS) sequence. Thus, it may be that the human host environment facilitated viral mutation, which resulted in insertion of this FCS sequence (10, 11). Mutations in the spike-protein region containing the FCS (^674^YQTQTNSPRRARSVASQ^690^) are common in SARS-CoV-2 variants, and certain mutations have been shown to enhance SARS-CoV-2 infectivity in mammalian model organisms and human cell lines (11). Interestingly, the FCS and flanking amino acid residues are similar in sequence to peptides found in venoms of elapid snakes, which include cobras and kraits, and marine cone snails (Table 1). The amino-acid sequence of the FCS and other similarities with α-neurotoxins led to the hypothesis that the spike protein, *via* the FCS, might interact with nicotinic acetylcholine receptors (nAChRs) and in particular subtypes that contain α7, α9, and/or α10 subunits (12, 13, 14). These subunits assemble to form pentameric complexes that are sensitive to inhibition by the snake peptide α-bungarotoxin and some α-conopeptides (14, 15, 16). Thus, sequence similarities among α-neurotoxins and the SARS-CoV-2 spike-protein formed the basis of a ‘nicotinic receptor hypothesis of COVID-19’ (17) which postulates that inhibition of immune cell-expressed nAChRs by the spike protein might contribute to the cytokine storm associated with severe COVID-19.

**Table 1:** Amino Acid Sequences of Several SARS-CoV-2 Spike-Protein Variants and Select α-Neurotoxins

Variant of interest	Sequence	Year of discovery	Reference
WT (B lineage)	^679^NSPRRAR↓SVASQ^690^	2019	(60)
Alpha (B.1.1.7)	^679^NSHRRAR↓SVASQ^690^	2020	(61)
Delta (B.1.617.2)	^679^NSRRRAR↓SVASQ^690^	2020	(61)
Omicron (B.1.1.529)	^679^KSHRRAR↓SVASQ^690^	2021	(61)
FLiRT (JN.1.7)	^679^KSRRRAR↓SVASQ^690^	2023	(62)
Neurotoxins			
α-Bungarotoxin	^29^CDVFCSSRGKV^39^	1963	(63)
α-Cobratoxin	^26^CDAFCSIRGKRV^37^	1990	(64)
α-Conopeptide RgIA	^1^GCCSDPRCRYRCR^13^	2008	(65)
α-Conopeptide ArIB	^1^DECCSNPACRVNNPHVCRRR^20^	2007	(29)

Wild type (WT); underlined residues indicate mutations from WT; the ‘↓’ denotes a potential cleavage site for the enzyme furin. The N-termini of the synthesized FCS peptides were acetylated and the C-termini amidated.

Human nAChRs are ligand-gated ion channels/receptors that assemble from the products of 16 individual genes (18). In most cases, nAChRs function as ion channels, but in non-neuronal cells nAChRs are non-ionic and function through other mechanisms (19). The nAChR subtypes implicated in the pathophysiology of COVID-19 include α7, α9∗, and α10∗ (the asterisk denotes the potential presence of other subunits). Importantly, these subunits are expressed by a variety of immune cells and control the release of proinflammatory cytokines. For example, inhibition of α7 and/or α9α10 nAChRs by α-conopeptides prevents the reduction of ATP-dependent release of the proinflammatory cytokine interleukin-1β (IL-1β) by acetylcholine (ACh) from human and mouse monocytes, macrophages, and lymphocytes (20, 21, 22, 23). Excessive release of proinflammatory cytokines has been associated with other respiratory diseases apart from COVID-19, including acute respiratory distress syndrome (ARDS) that can progress to cytokine release syndrome (CRS) and/or sepsis (24).

Testing the nicotinic receptor hypothesis of COVID-19 has been hampered by the lack of spike-protein constructs that are suitable for use in live cell experiments in large part due to the hydrophobicity of the spike protein itself necessitating high concentrations of organic solvents and detergents to maintain the protein in solution. Additionally, the spike protein has been suggested to possess other nAChR-binding domains apart from the FCS complicating identification of specific residues responsible for activity (13). Furthermore, many commercially available spike-proteins purposefully lack critical amino acids of the FCS to keep the protein intact when exposed to or expressed in mammalian cells that have enzymes such as furin. Specifically, key residues of the spike protein (^682^RRAR^685^) are often replaced by sequences, for example ^682^SRAS^685^, that render the FCS insensitive to proteolytic cleavage and preserve the spike protein intact. Consequently, such constructs are not suitable for testing potential FCS interactions with nAChRs (14). Non-commercial peptides have been generated that mimic the spike protein, but these constructs generally had low potency (IC_50_s in the high μM range) or no inhibitory activity on α7 and/or α9α10 nAChRs (12, 25) calling into question the role of nAChRs in COVID-19. In this work, we synthesized a series of short peptides corresponding to the FCSs of WT (wild-type; lineage B), Alpha (B.1.1.7), Delta (B.1.617.2), Omicron (B.1.1.529), and FLiRT (JN.1.7) SARS-CoV-2 variants to isolate and identify FCS interactions with nAChRs. We show that several of these peptides are potent inhibitors of human α7, α9, α10, and α9α10 nAChRs. The inhibitory effects of the five SARS-CoV-2 peptides were both virus variant and nAChR-subtype dependent. Furthermore, using human monocytic THP-1 cells, we show that these peptides prevent ACh-mediated inhibition of ATP-induced release of IL-1β, a key inflammatory cytokine. Our results suggest that SARS-CoV-2 might have evolved to include mutations of the FCS that enhance inhibitory activity on nAChRs.

## Results

### Peptides containing residues of the SARS-CoV-2 spike-protein FCS are antagonists of nAChRs

**Figure 1:**
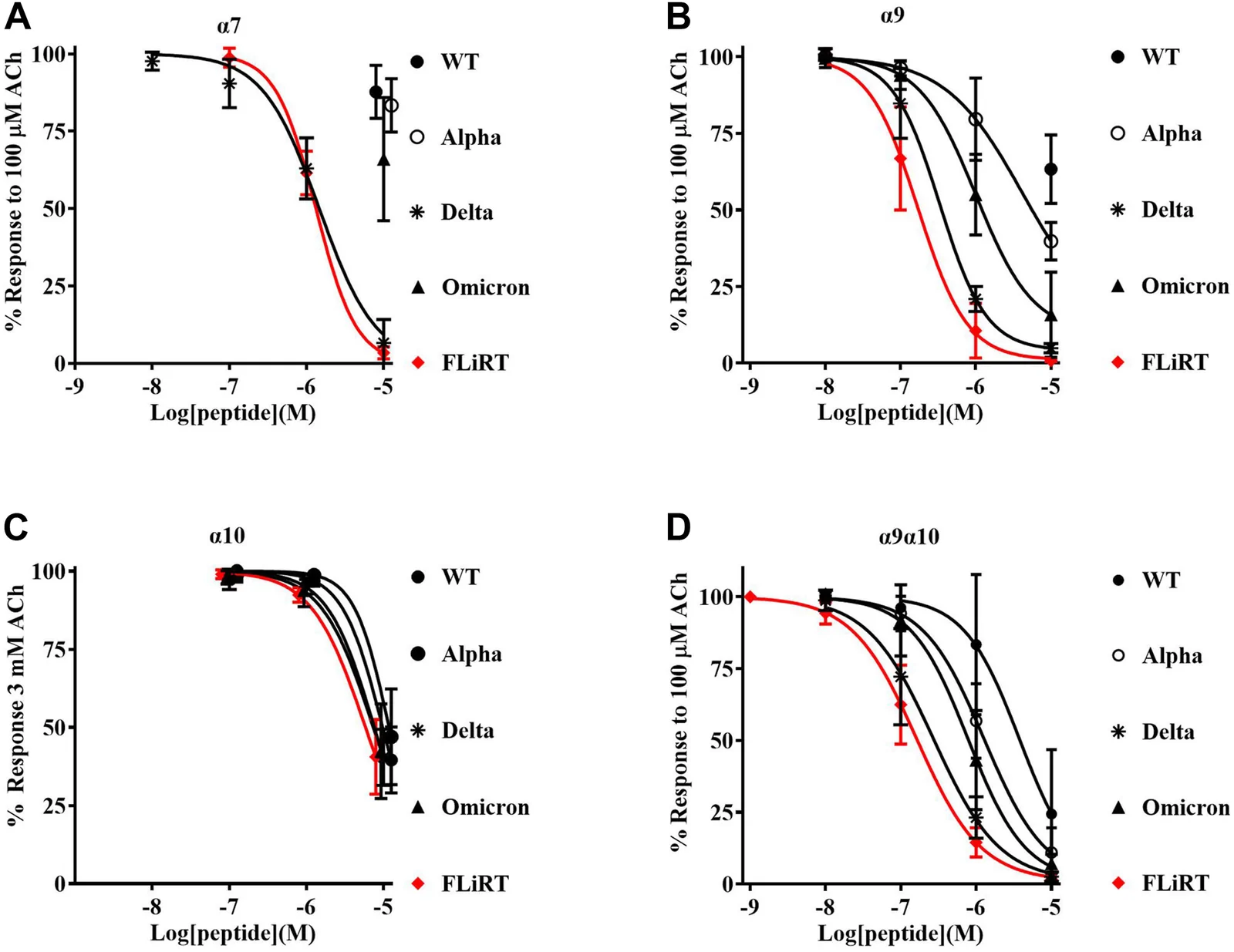
**Furin cleavage-site (FCS) peptides are antagonists of α7 and α9/α10 nAChRs.***X. laevis* oocytes were used to assess the ability of FCS peptides to inhibit ACh-evoked currents mediated by the indicated nAChR subtypes as described in Experimental procedures. *A*, concentration-response analysis demonstrated that peptides corresponding to the Delta and FLiRT FCSs inhibited α7 nAChRs in the nM to low μM range. By contrast, the IC_50_ values for WT, Alpha, and Omicron peptides were >10 μM; the ACh-evoked responses were 88 ± 9%, 83 ± 9%, and 66 ± 20% of control responses in the presence of FCS peptides (10 μM), respectively. *B*, concentration-response analysis of FCS peptide inhibition of α9 nAChRs. Peptides corresponding to the Alpha, Delta, Omicron, and FLiRT FCSs inhibited α9 nAChRs in the nM to low μM range. The WT peptide had an IC_50_ value > 10 μM; the ACh-evoked responses were 66 ± 13% of control responses in the presence of the peptide (10 μM). *C,* concentration-response analysis of FCS peptide inhibition of α10 nAChRs. All five FCS peptides had similar IC_50_ values (∼10 μM). *D*, concentration-response analysis of FCS peptide inhibition of heteromeric α9α10 nAChRs. All five FCS peptides had IC_50_ values < 10 μM, but values corresponding to Delta and FLiRT were the lowest. The error bars represent the SD of the data from ≥5 individual oocytes. See Table 2 for IC_50_ values.

We synthesized peptides containing the FCS sequences of five SARS-CoV-2 variants (Table 1). The N-termini were acetylated and the C-termini amidated to avoid positive and negative charges, respectively, that would be absent in native uncleaved spike-protein. The resulting FCS peptides were assessed for activity on nAChRs using two-electrode voltage-clamp (TEVC) electrophysiology of *Xenopus laevis* oocytes expressing human homomeric α7, α9, or α10 nAChRs. The peptides were also tested on heteromeric human α9α10 nAChRs. Figure 1 shows the concentration-response analyses of peptide activity on the indicated nAChR subtypes. The α7 nAChR subtype was sensitive to inhibition by the Delta and FLiRT (nM IC_50_s) peptides but not to WT, Alpha, or Omicron (IC_50_s > 10 μM; Fig. 1*A*). α9 nAChR homomers were also potently inhibited by Delta and FLiRT (nM IC_50_s) but were less sensitive to inhibition by the WT, Alpha, and Omicron variants (IC_50_ values 1–10 μM; Fig. 1*B*). All five peptides inhibited α10 homomers with IC_50_s in the ∼10 μM range (Fig. 1*C*). In contrast to the results found for α7, α9, and α10 homomers, all five peptides had IC_50_s in the high nM to low μM range for α9α10 heteromers including the WT variant which showed less inhibitory activity on the three homomeric subtypes (Fig. 1*D*). The IC_50_ values for all peptide-receptor combinations are provided in Table 2. These data indicate that spike-protein peptides can potently interact with nAChRs with affinity dependent on virus variant and nAChR subtype.

**Table 2:** Concentration-Response Analysis of FCS Peptide Potency for Inhibition of nAChRs

nAChR subtype	α7	n	α9	n	α10	n	α9α10	n
FCS Peptide								
WT	>10 μM	7	>10 μM	4	7.76 (6.46–9.32) μM	5	3.75 (1.91–7.39) μM	5
Alpha	>10 μM	6	5.85 (4.33–7.96) μM	5	9.40 (7.52–11.8) μM	5	1.28 (0.982–1.66) μM	5
Delta	1.46 (1.14–1.89) μM	5	331 (247–443) nM	4	7.62 (6.49–9.01) μM	5	272 (197–376) nM	5
Omicron	>10 μM	6	1.01 (0.568–1.78) μM	5	7.91 (6.02–10.4) μM	5	782 (565–1083) nM	5
FLiRT	1.33 (1.16–1.52) μM	5	177 (131–238) nM	5	7.37 (5.95–9.13) μM	5	163 (126–211) nM	5

Values in parentheses indicate the 95% CI of the estimated IC_50_ value; the ‘n’ denotes the number of individual oocytes assessed.

### Furin cleavage-site peptides prevent ACh-mediated inhibition of BzATP-evoked IL-1β release from human monocytic THP-1 cells

**Figure 2:**
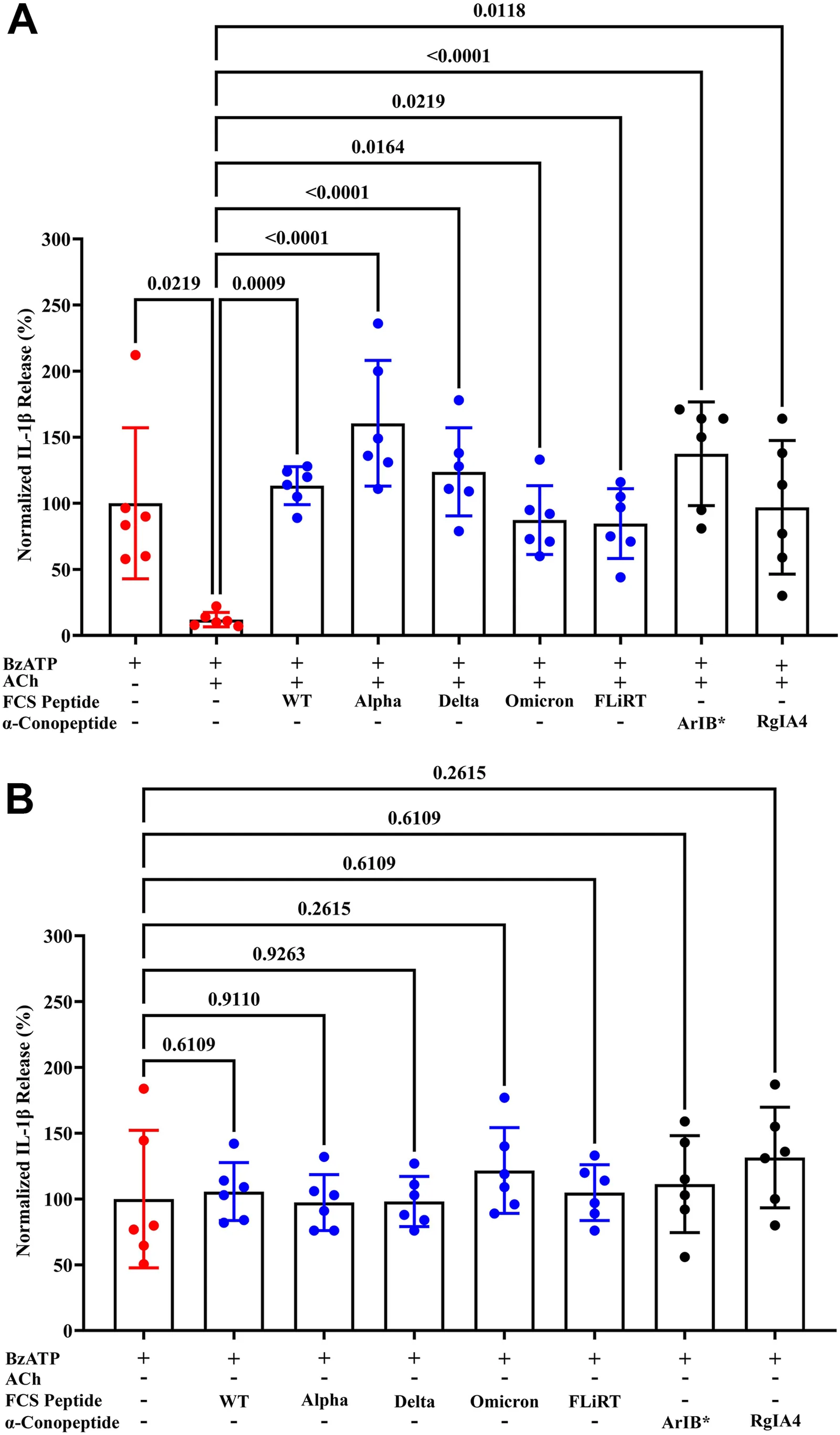
**Furin cleavage-site (FCS) peptides prevent ACh-mediated inhibition of BzATP-evoked IL-1β release from monocytic THP-1 cells.** THP-1 monocytes were primed with LPS (1 μg/ml, 5 h) then stimulated with BzATP (100 μM; 40 min) to evoke the release of IL-1β that was quantified by ELISA as described in Experimental procedures. *A*, the ability of the FCS peptides (10 μM) to prevent the inhibitory effect of ACh (100 μM) on the BzATP-induced IL-1β release was assessed. All five peptides as well as the α7 nAChR antagonist α-conopeptide ArIB∗ and the α9α10 nAChR antagonist α-conopeptide RgIA4 prevented the inhibitory effect of ACh. The amount of BzATP-evoked IL-1β released in the presence of ACh was normalized to the amount evoked by BzATP in the absence of ACh. *B*, BzATP-evoked IL-1β release in the absence of ACh and in the presence of FCS peptides. No effect of FCS peptides or α-conopeptides on the BzATP-induced IL-1β release was found in the absence of ACh. The error bars represent the SD of the data from six individual biological replicates. ArIB∗, [V11L,V16D]ArIB. The data were analyzed for significance using a Friedman test followed by a BKY *post hoc* test. Results were considered a discovery if q ≤ 0.05; see Table 3 for a summary of the results.

In order to determine the effects of FCS peptides on the release of cytokines from immune cells, we used human monocytic THP-1 cells that are known to express several nAChR subtypes (26, 27). These cells respond to the purinergic receptor agonist BzATP by releasing the proinflammatory cytokine IL-1β (27). THP-1 cells were seeded in 48 well plates and incubated with lipopolysaccharide (LPS; 1 μg/ml) for 5 hours prior to stimulation with BzATP (100 μM/40 min). Figure 2*A* shows that all five FCS peptides (10 μM) prevented ACh (100 μM) from inhibiting BzATP-evoked IL-1β release. The FCS peptides themselves had no effect on BzATP-evoked IL-1β release in the absence of ACh (Fig. 2*B*). Experiments were also performed to determine if the FCS peptides alone had any inherent activity on IL-1β release. Under these conditions, the amount of IL-1β released in the control group (LPS only) relative to the amount released in the presence of FCS peptide was similar (Fig. S1). For comparison, the α7 antagonist α-conopeptide [V11L,V16D]ArIB (ArIB∗) and the α9∗ antagonist α-conopeptide RgIA4 were also assessed and found to prevent ACh-mediated inhibition of IL-1β release (Fig. 2*A*) in line with previous data (21, 22). Similar results were found when a lower concentration (3 μM) of the FCS peptides was tested under these conditions (Fig. S2, *A* and *B*). The results of Figure 2 are summarized in Table 3 and indicate that the spike-protein region containing the FCS may modulate the activity of immune cells by inhibiting the anti-inflammatory effects of ACh *via* receptors that contain α7, α9, and/or α10 subunits.

**Table 3:** BzATP-Evoked IL-1β Release From Monocytic THP-1 Cells

Experimental condition	Normalized IL-1β release (%)	
	BzATP + ACh	BzATP–ACh
BzATP	100 ± 57	100 ± 52
BzATP + ACh	12 ± 6	-
WT	113 ± 14	106 ± 22
Alpha	161 ± 48	97 ± 21
Delta	124 ± 33	98 ± 19
Omicron	87 ± 26	122 ± 33
FLiRT	85 ± 26	105 ± 21
ArIB∗	138 ± 39	111 ± 37
RgIA4	97 ± 51	132 ± 38

All conditions were performed in the presence of LPS (1 μg/ml). The ‘±’ represents the SD of the data obtained from 6 individual experiments; ArIB∗, [V11L,V16D]ArIB. The BzATP + ACh column corresponds to Figure 2*A* and the BzATP–ACh column to Figure 2*B*.

### Quantitative real-time PCR analysis of nAChR expression in monocytic THP-1 cells

**Figure 3:**
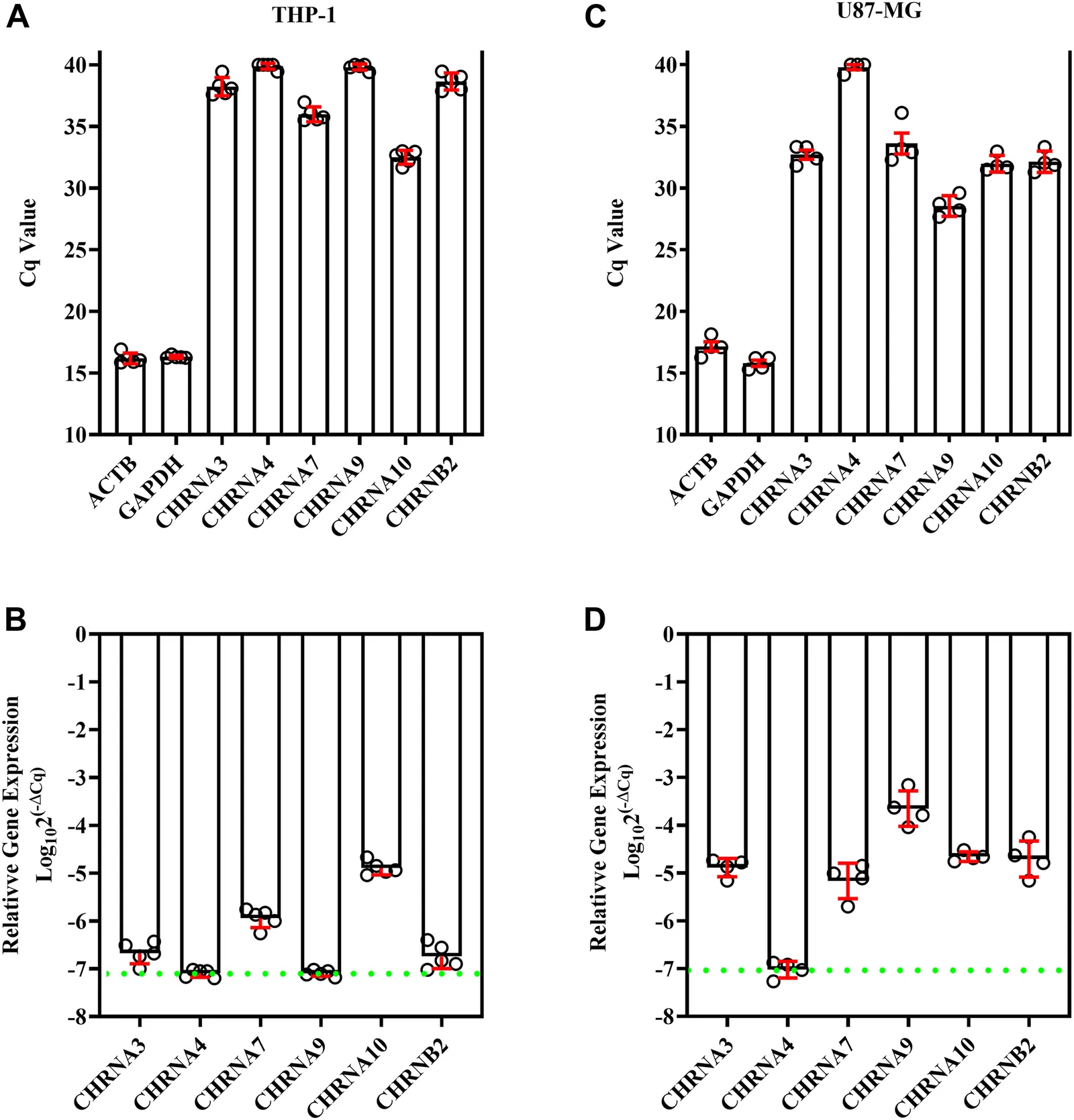
**Quantitative PCR experiments reveal the presence of RNA for several nAChR genes in THP-1 and U87-MG cells.***A*, C_q_ values obtained for ACTB, GAPDH, and nAChR subunit genes expressed by THP-1 cells. RNA was detected for CHRNA3, CHRNA7, CHRNA10, and CHRNB2. RNA levels for CHRNA4 and CHRNA9 were below the threshold of detection (C_q_ values ≥ 40). *B*, the relative expression of nicotinic subunit genes in THP-1 cells was determined by normalizing the nicotinic subunit gene C_q_ values to those for ACTB and GAPDH as described in Experimental procedures. *C*, control experiments were performed by probing the expression of nicotinic subunit genes in U87-MG cells. RNAs were detected for CHRNA3, CHRNA7, CHRNA9, CHRNA10, and CHRNB2. Levels of CHRNA4 were below the level of detection (C_q_ values ≥ 40). *D*, the relative expression of nicotinic subunit genes in U87-MG cells. The error bars in A-D indicate the SD obtained from five individual biological replicates for THP-1 cells and four for U87-MG cells. The green dotted lines in *B* and *D* indicate a corresponding C_q_ value of ∼40. See Table 4 for C_q_ and relative expression values.

THP-1 cells are known to express nAChRs that are sensitive to subtype-selective agonists and antagonists of the α7 and α9∗ receptors (26, 27). However, very little information has been reported on the expression of other nAChR subunits. To compare the relative expression levels of nAChR subunit genes in THP-1 cells, we performed qPCR and assessed the cells for the presence of mRNAs for α7, α9, and α10 as well as α3, α4, and β2 subunits. Figure 3*A* shows the C_q_ values for the six nAChR genes assessed as well as those for the reference genes ACTB (β-actin) and GAPDH (glyceraldehyde-3-phosphate dehydrogenase). Figure 3*B* shows the log_10_^2-(ΔCq)^ values for the nAChR subunit genes normalized to the reference gene C_q_ values. Of the six genes analyzed, transcripts for CHRNA7 and CHRNA10 were the most abundant followed by CHRNA3 and CHRNB2, whereas transcripts for CHRNA9 were variably detected and, in some samples, were below the threshold level of detection (C_q_ value ≥ 40). Transcripts for CHRNA4 were not detected under the conditions used in this study. As a control, we assessed a second cell line, human glioblastoma U87-MG cells, known to express α9 and α10 nAChRs (28). The most abundant transcripts detected in U87-MG cells were for CHRNA9 followed by CHRNA3, CHRNA7, CHRNA10, and CHRNB2 (Fig. 3, *C* and *D*). Transcripts for CHRNA4 were below the level of detection under these conditions. The C_q_ and normalized expression values for each sample/gene combination are provided in Table 4.

**Table 4:** qPCR Analysis of nAChR Subunit Gene Expression in Human THP-1 Monocytes and U87-MG Cells

Gene	THP-1			U87-MG		
	C_q_	n	log_10_2^-(ΔC^^q^^)^	C_q_	n	log_10_2^-(ΔC^^^p^^^)^
*ACTB*	16.2 ± 0.4	5	nd	17.2 ± 0.8	4	nd
*GAPDH*	16.3 ± 0.1	5	nd	15.8 ± 0.5	4	nd
*CHRNA3*	38.2 ± 0.7	5	−6.8 ± 0.2	32.7 ± 0.7	4	−4.9 ± 0.2
*CHRNA4*	39.9 ± 0.2	5	−7.1 ± 0.1	39.8 ± 0.5	4	−7.0 ± 0.2
*CHRNA7*	36.0 ± 0.2	5	−5.9 ± 0.2	33.6 ± 1.7	4	−5.2 ± 0.4
*CHRNA9*	39.9 ± 0.1	5	−7.1 ± 0.1	28.6 ± 0.8	4	−3.7 ± 0.4
*CHRNA10*	32.5 ± 0.6	5	−4.9 ± 0.1	32.0 ± 0.7	4	−4.7 ± 0.4
*CHRNB2*	38.6 ± 0.7	5	−6.7 ± 0.3	32.1 ± 0.9	4	−4.6 ± 0.3

The ‘±’, represents SD; the ‘n’ denotes the number of individual experiments; ‘nd’, indicates values not determined.

### Furin cleavage-site peptides, α-conopeptides ArIB∗, RgIA4, and PeIA∗ show nAChR subtype selectivity

**Figure 4:**
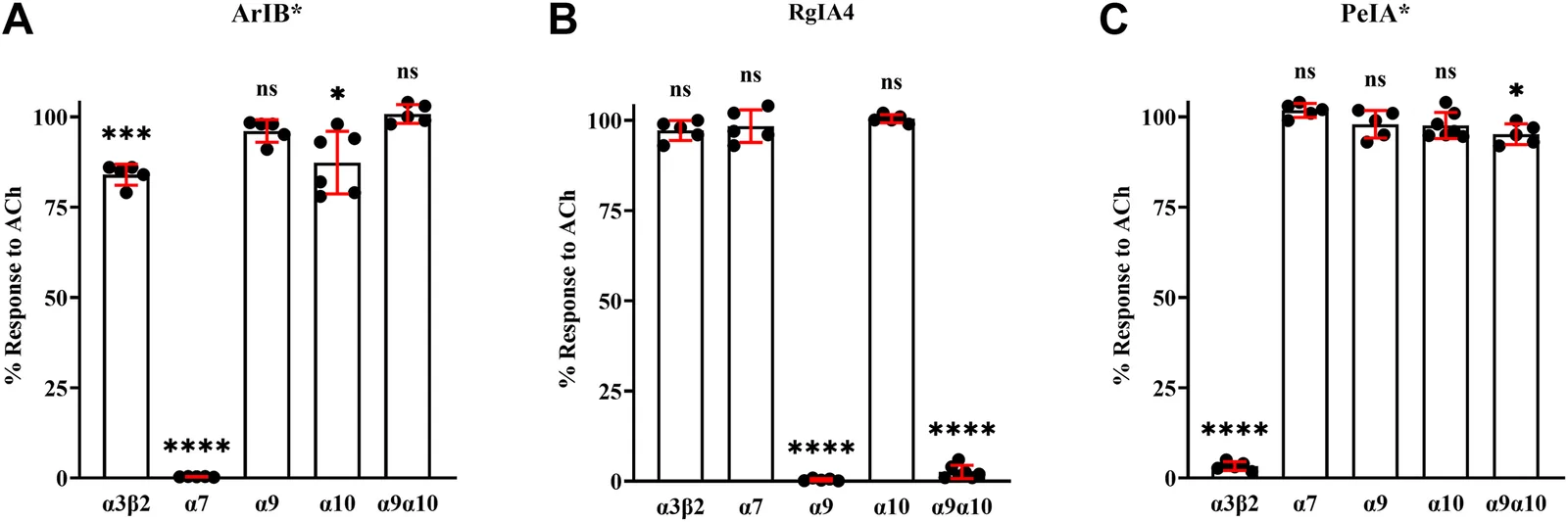
**Select α-conopeptides distinguish among α3β2, α7, α9, α10, and α9α10 nAChR subtypes.***X. laevis* oocytes were used to assess the ability of ArIB∗, RgIA4, and PeIA∗ to selectively antagonize the nAChR subtypes potentially expressed in monocytic THP-1 cells as described in Experimental procedures. *A*, ArIB∗ (500 nM) inhibited responses to ACh mediated by α7 nAChRs and showed minimal inhibition of α3β2 and α10. The peptide was inactive on α9 and α9α10 subtypes. *B*, RgIA4 (200 nM) inhibited responses mediated by α9 and α9α10 nAChRs but was inactive on α3β2, α7, and α10 subtypes. *B*, PeIA∗ (10 nM) inhibited responses to ACh mediated by α3β2 nAChRs and was essentially inactive on all other subtypes tested. The error bars indicate the SD of the data from at ≥5 individual biological replicates. Significance was determined using a two-tailed, one-sample *t* test and compared to a hypothetical response value of 100% *i.e.*, no effect; ∗*p* ≤ 0.05, ∗∗*p* ≤ 0.01, ∗∗∗*p* ≤ 0.001, ∗∗∗∗*p* ≤ 0.0001; see Table 5 for individual response and *p* values. ArIB∗, [V11L,V16D]ArIB; PeIA∗, [S9H,V10A,E14N]PeIA.

**Figure 5:**
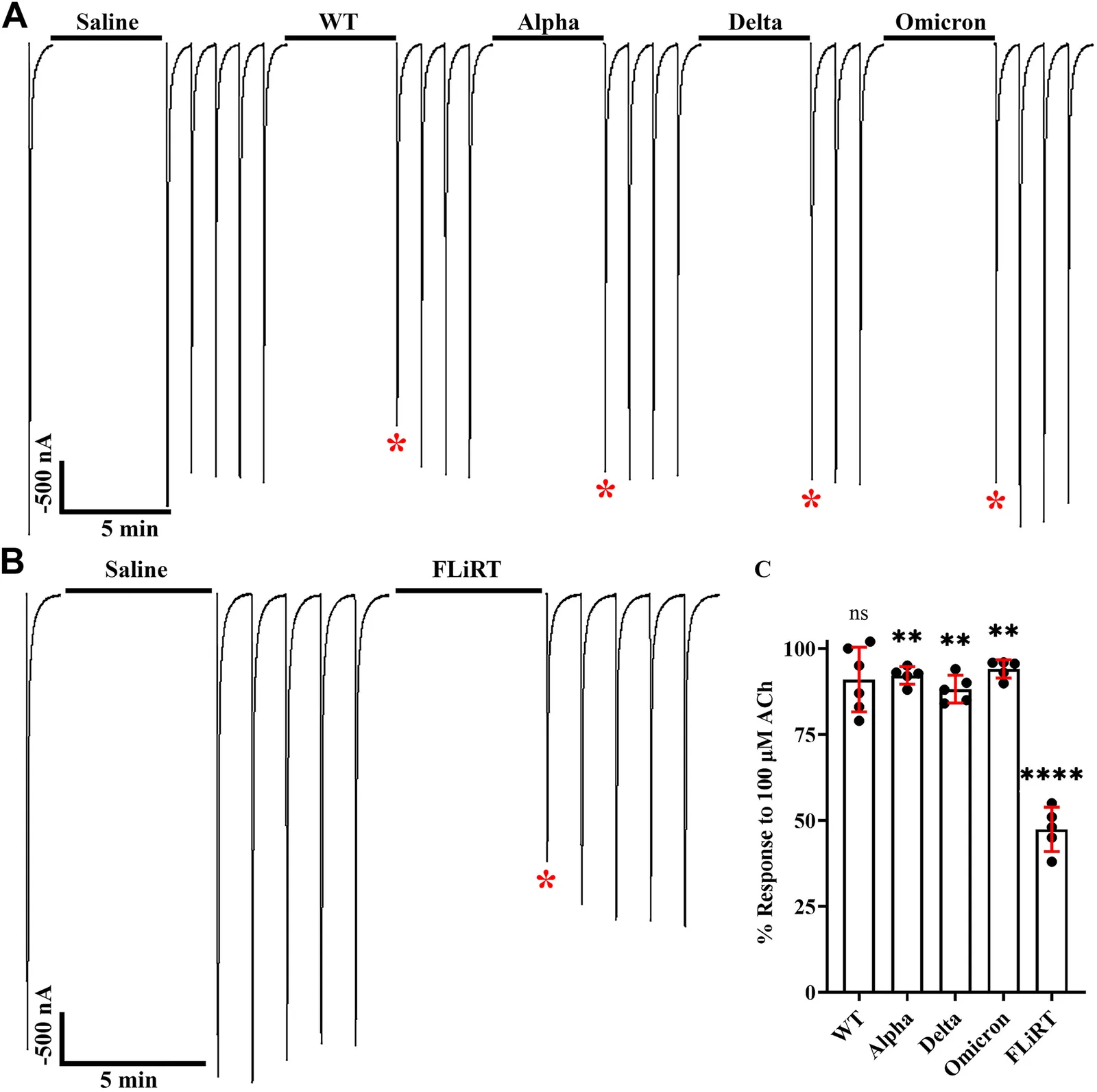
**Furin cleavage-site (FCS) peptides inhibit human α3β2 nAChRs.***X. laevis* oocytes were used to assess the ability of FCS peptides to inhibit ACh-evoked currents mediated by α3β2 nAChRs as described in Experimental procedures. *A*, traces of ACh-evoked currents before, during, and after a 5-min exposure to the indicated FCS peptide (3 μM); the responses in the presence of the WT, Alpha, Delta, and Omicron FCS peptides were 91 ± 9% (*p* = 0.660), 92 ± 3% (*p* = 0.0024), 88 ± 4% (*p* = 0.0028), and 94 ± 3% (*p* = 0.0073), respectively, of control values. *B*, traces of ACh-evoked currents before, during, and after a 5-min exposure to the FLiRT FCS peptide (3 μM). The ACh-evoked responses were 47 ± 6% (*p* < 0.0001) of control values. *C*, scatter plot summary of the values obtained in A and B. The error bars in C represent the SD of the data obtained from ≥5 individual oocytes. All current traces in *A* and *B* were obtained from the same oocyte and shown concatenated except for the 5-min gaps when saline or the indicated peptide was applied. The *red asterisks* indicate the response to ACh after a 5 min static bath exposure to the FCS peptides. Significance was determined using a two-tailed, one-sample *t* test and compared to a hypothetical response value of 100% *i.e.*, no effect; ∗*p* ≤ 0.05, ∗∗*p* ≤ 0.01, ∗∗∗*p* ≤ 0.0001, ∗∗∗∗*p* ≤ 0.0001.

The qPCR results indicated that THP-1 cells may express nAChRs composed of α7 and/or α10 subunits and potentially subtypes containing α3, α9, and/or β2 subunits. To pharmacologically dissect the presence of THP-1 nAChRs, we selected three α-conopeptides that distinguish among nAChR containing α3, α7, and α9/α10 subunits. α-Conopeptides ArIB∗ and RgIA4 are selective antagonists of rat α7 and α9α10 nAChRs, respectively (29, 30), and PeIA∗ is a potent antagonist of rat α3β2 nAChR (31). To ensure that these α-conopeptides maintained subtype selectivity for human nAChRs and could be used to pharmacologically identify the receptors of interest, we tested each of them on human α3β2, α7, α9, α10, and α9α10 expressed in *Xenopus* oocytes (Fig. 4 and Table 5). ArIB∗ completely inhibited the α7 subtype but showed minimal inhibition of α3β2 and α10 nAChRs (Fig. 4*A*). α9 homomers and α9α10 heteromers were insensitive to the peptide. By contrast, the α9∗-selective peptide RgIA4 completely inhibited α9 homomers and α9α10 heteromers, but α7 and α3β2 nAChRs were insensitive to the peptide (Fig. 4*B*). PeIA∗ inhibited ∼98% of the ACh-evoked responses mediated by α3β2 nAChRs but showed very little to no activity on α7, α9, α10 or α9α10 subtypes (Fig. 4*C*). Thus, ArIB∗, RgIA4 and PeIA∗ can be used to pharmacologically distinguish among α7, α9∗, and α3β2 nAChR subtypes, respectively. Next, we assessed the activity of the FCS peptides on α3β2 nAChRs. Figure 5 shows traces of ACh-evoked currents recorded from an oocyte expressing human α3β2 nAChRs. The WT, Alpha, Delta, and Omicron peptides showed very little inhibitory activity at the concentration tested (3 μM) (Fig. 5*A*). However, the FLiRT peptide inhibited ∼50% of the ACh-evoked currents at the same concentration (Fig. 5*B*). A scatter plot summary of the data is presented in Figure 5*C*. These results indicate that the sequence of a given SARS-CoV-2 FCS may lead to differential effects depending on the nAChR subtype present.

**Table 5:** Potency of α-conopeptides ArIB∗, RgIA4, and PeIA∗ for inhibition of nAChRs

α-Conopeptide	nAChR subtype									
	α3β2		α7		α9		α10		α9α10	
	% Response	n	%Response	n	%Response	n	%Response	n	%Response	n
α-conopeptide										
ArIB∗	84 ± 3 (*p* = 0.0003)	5	0.3 ± 0.1 (*p* < 0.0001)	5	96 ± 3 (*p* = 0.0501)	5	87 ± 9 (*p* = 0.0159)	6	101 ± 3 (*p* = 0.5275)	5
RgIA4	97 ± 3 (*p* = 0.0870)	5	98 ± 5 (*p* = 0.4716)	5	0.4 ± 0.1 (*p* < 0.0001)	5	100 ± 1 (*p* = 4307)	5	3 ± 2 (*p* < 0.0001)	7
PeIA∗	3 ± 1 (*p* < 0.0001)	5	102 ± 2 (*p* = 0.1045)	5	98 ± 4 (*p* = 0.2986)	5	98 ± 4 (*p* = 0.1342)	7	95 ± 3 (*p* = 0.0200)	5

Values are expressed as ‘percent response’ of control values, and the ± represents the SD; the ‘n’ denotes the number of oocytes assessed; ArIB∗, [V11L,V16D]; PeIA∗, [S9H,V10A,E14N]PeIA.

**Table 6:** BzATP-Evoked IL-1β Release From Monocytic THP-1 Cells

Experimental condition	Normalized IL-1β release (%)	
	BzATP + ACh	BzATP–ACh
BzATP	100 ± 44	-
BzATP + ACh	15 ± 11	-
FLiRT (3 μM)	120 ± 28	86 ± 23
FLiRT (10 μM)	93 ± 42	93 ± 15
ArIB∗	85 ± 23	90 ± 18
RgIA4	83 ± 26	88 ± 41
PeIA∗	29 ± 31	114 ± 27

**Figure 6:**
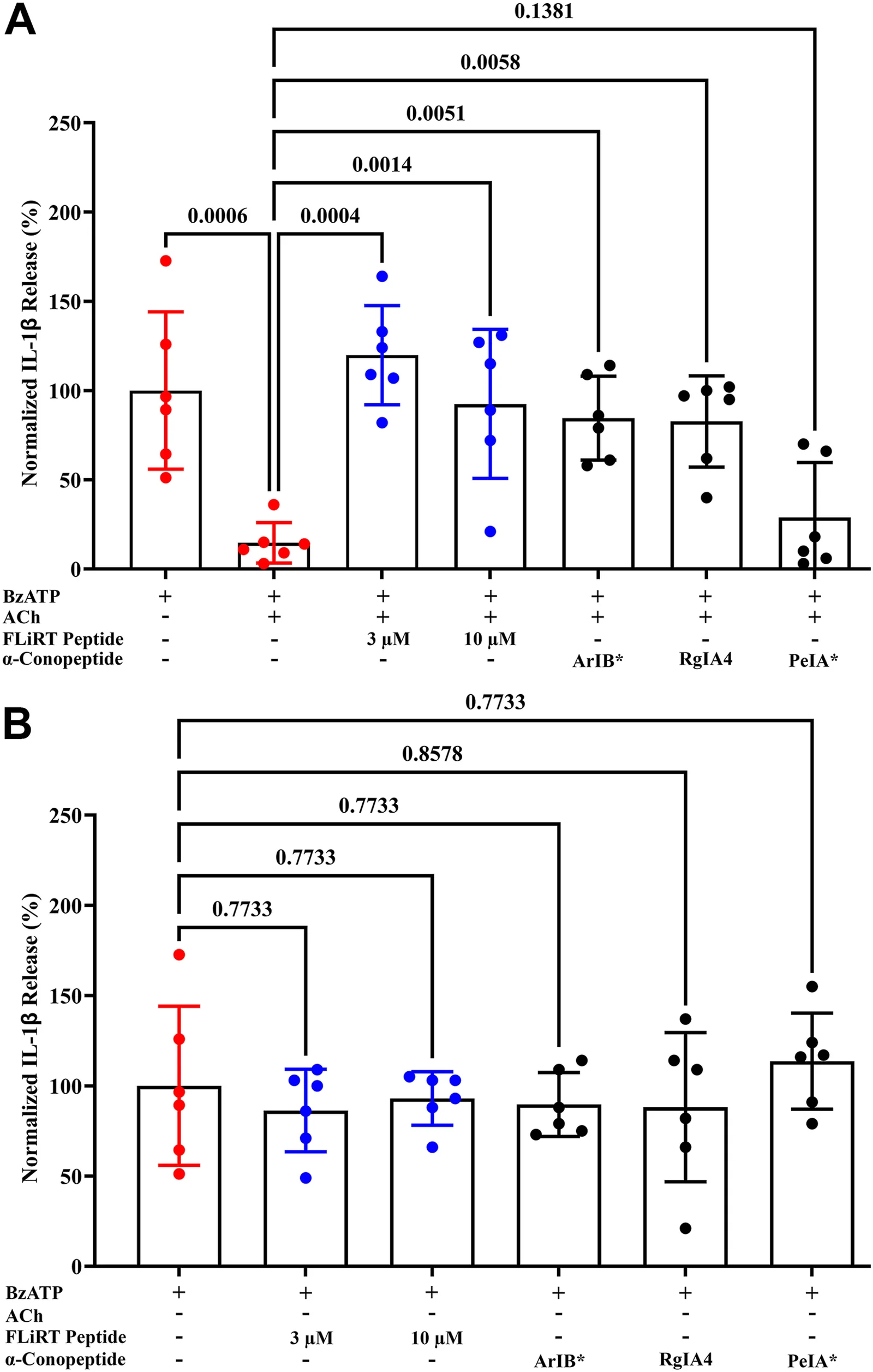
**Peptides that target α7 and α9α10 nAChRs, but not the α3β2 subtype, prevent ACh-mediated inhibition of BzATP-evoked IL-1β release from monocytic THP-1 cells.** Monocytic THP-1 cells were exposed to LPS (1 μg/ml, 5 h) then stimulated with BzATP for 40 min to evoke the release of IL-1β and quantified by ELISA as described in Experimental procedures. *A*, the impact of the FLiRT peptide (3 μM and 10 μM) on the ability of ACh (100 μM) to inhibit BzATP-mediated IL-1β release was assessed. The amount of BzATP-evoked IL-1β released in the presence of ACh was normalized to the amount evoked by BzATP in the absence of ACh. Peptide effect was assessed for significance by comparing the amount of IL-1β obtained in the presence of the peptide to that obtained in the BzATP + ACh group. IL-1β release was also assessed in the presence of the α7 nAChR antagonist α-conopeptide ArIB∗ (100 nM), the α9α10 nAChR antagonist α-conopeptide RgIA4 (200 nM), or the α3β2 antagonist α-conopeptide PeIA∗ (10 nM). The error bars represent the SD of the data obtained from 6 individual biological replicates. The data were analyzed for significance using a Friedman test followed by a BKY *post hoc* test. Results were considered a discovery if q ≤ 0.05; see Table 6 for a summary of the results. ArIB∗, [V11L,V16D]ArIB; PeIA∗, [S9H,V10A,E14N]PeIA.

All experiments were performed in the presence of LPS (1 μg/ml). The ‘±’ represents the SD of the data obtained from 6 individual samples; ArIB∗, [V11L,V16D]ArIB; PeIA∗, [S9H,V10A,E14N]PeIA. The BzATP + ACh column corresponds to Figure 6*A* and the BzATP–ACh column to Figure 6*B*.

**Table 7:** BzATP-Evoked IL-1β Release From Monocytic THP-1 Cells

Experimental condition	Normalized IL-1β release (%)	
	BzATP + ACh	BzATP–ACh
BzATP	100 ± 56	-
BzATP + ACh	16 ± 8	-
ArIB∗ (100 nM)	103 ± 14	79 ± 18
RgIA4 (200 nM)	87 ± 17	83 ± 16
OmIA (10 μM)	106 ± 13	84 ± 18

**Figure 7:**
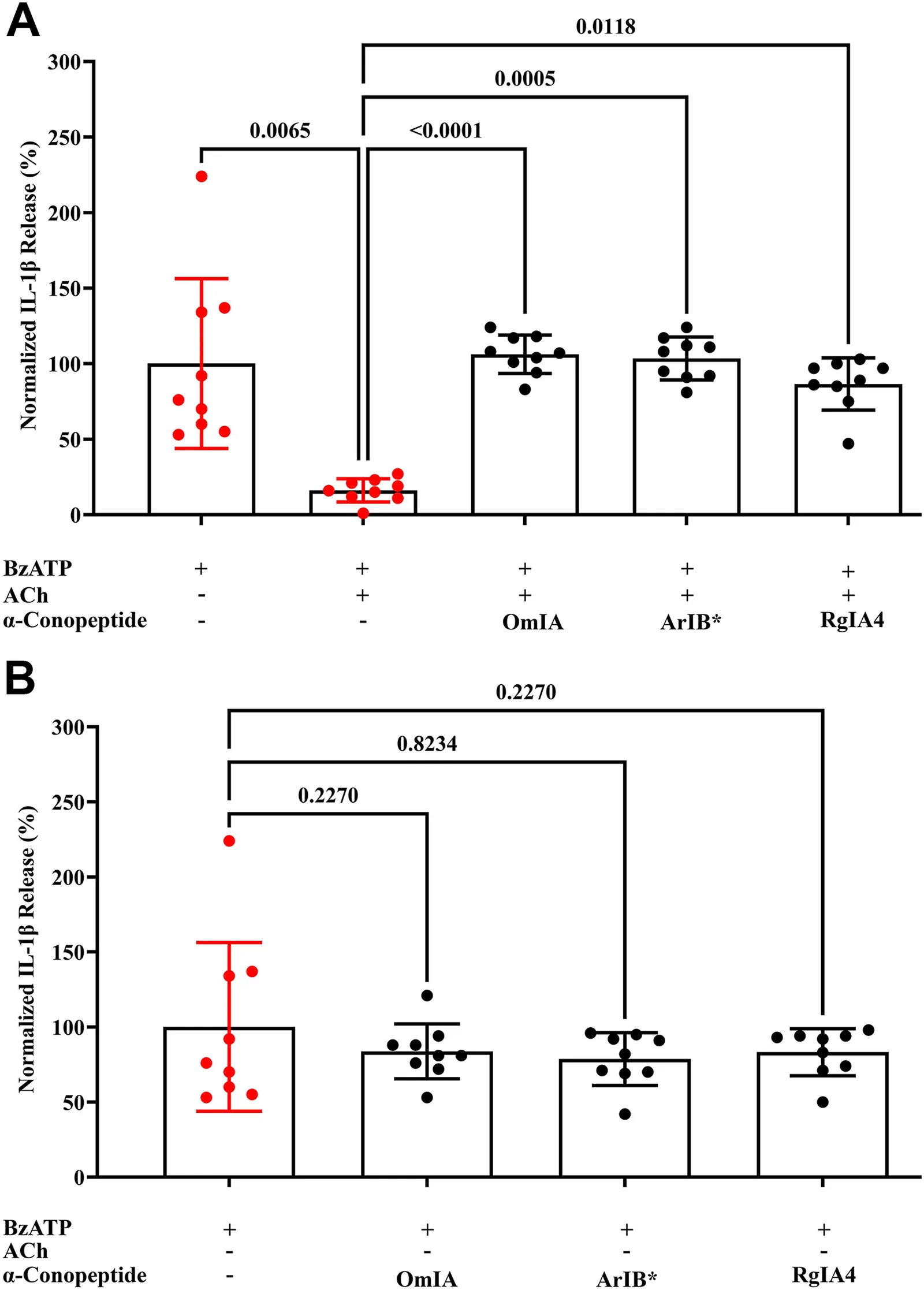
**α-Conopeptides OmIA, ArIB∗, and RgIA4 prevent ACh-mediated inhibition of BzATP-evoked IL-1β release from monocytic THP-1 cells.** THP-1 monocytes were primed with LPS (1 μg/ml, 5 h) then stimulated with BzATP (100 μM; 40 min) to evoke the release of IL-1β that was quantified by ELISA as described in Experimental procedures. *A,* the ability of OmIA, ArIB∗, and RgIA4 to prevent the inhibitory effect of ACh (100 μM) on the BzATP-induced IL-1β release was assessed. All three α-conopeptides reversed the inhibitory effect of ACh. The amount of BzATP-evoked IL-1β released in the presence of ACh was normalized to the amount evoked by BzATP in the absence of ACh. *B*, BzATP-evoked IL-1β release in the absence of ACh and in the presence α-conopeptides; no effect on BzATP-induced IL-1β release was found in the absence of ACh. The error bars represent the SD of the data from 9 individual biological replicates. ArIB∗, [V11L,V16D]ArIB. The data were analyzed for significance using a Friedman test followed by a BKY *post hoc* test. Results were considered a discovery if q ≤ 0.05; see Table 7 for a summary of the results.

All experiments were performed in the presence of LPS (1 μg/ml). The ‘±’ represents the SD of the data obtained from 9 individual samples; ArIB∗, [V11L,V16D]ArIB. The BzATP + ACh column corresponds to Figure 7*A* and the BzATP–ACh column to Figure 7*B*.

### α-Conopeptides ArIB∗, OmIA, and RgIA4, but not PeIA∗, prevent ACh-mediated inhibition of BzATP-evoked IL-1β release from human monocytic THP-1 cells.

ArIB∗, RgIA4, and PeIA∗ were tested for their ability to inhibit the effect of BzATP stimulated IL-1β release from THP-1 monocytes. In parallel experiments, we also reassessed the effects of the FLiRT peptide for comparison with the α-conopeptides. Similar to the results presented in Figure 2, ArIB∗ and RgIA4 completely inhibited the effects of ACh on IL-1β release (Fig. 6*A*). PeIA∗, however, was without effect. Results obtained with the FLiRT FCS peptide in the absence of ACh (Fig. 6*B*) were also similar to those obtained previously (Fig. 2*B*). Neither the FLiRT peptide nor the α-conopeptides had any direct effects on IL-1β release (Fig. S3). Finally, to confirm the lack of potential off-target effects on IL-1β release that might occur from Arg rich peptides, we tested a fourth α-conopeptide, OmIA, that lacks Arg residues but nevertheless shows nM potency for α7 nAChRs (32). OmIA was tested on oocytes expressing human α7 nAChRs and inhibited ACh-mediated responses with an IC_50_ value of 238 nM (Fig. S4). By contrast, human α9α10 nAChRs shows very little inhibition at the maximal OmIA concentration tested (10 μM). These results further confirm that the nAChR subtypes that control IL-1β release from THP-1 cells contain α7 and α9/α10 subunits (20, 21, 22) and that the α3β2 subtype is unlikely to be involved to a significant degree.

## Discussion

**Figure 8:**
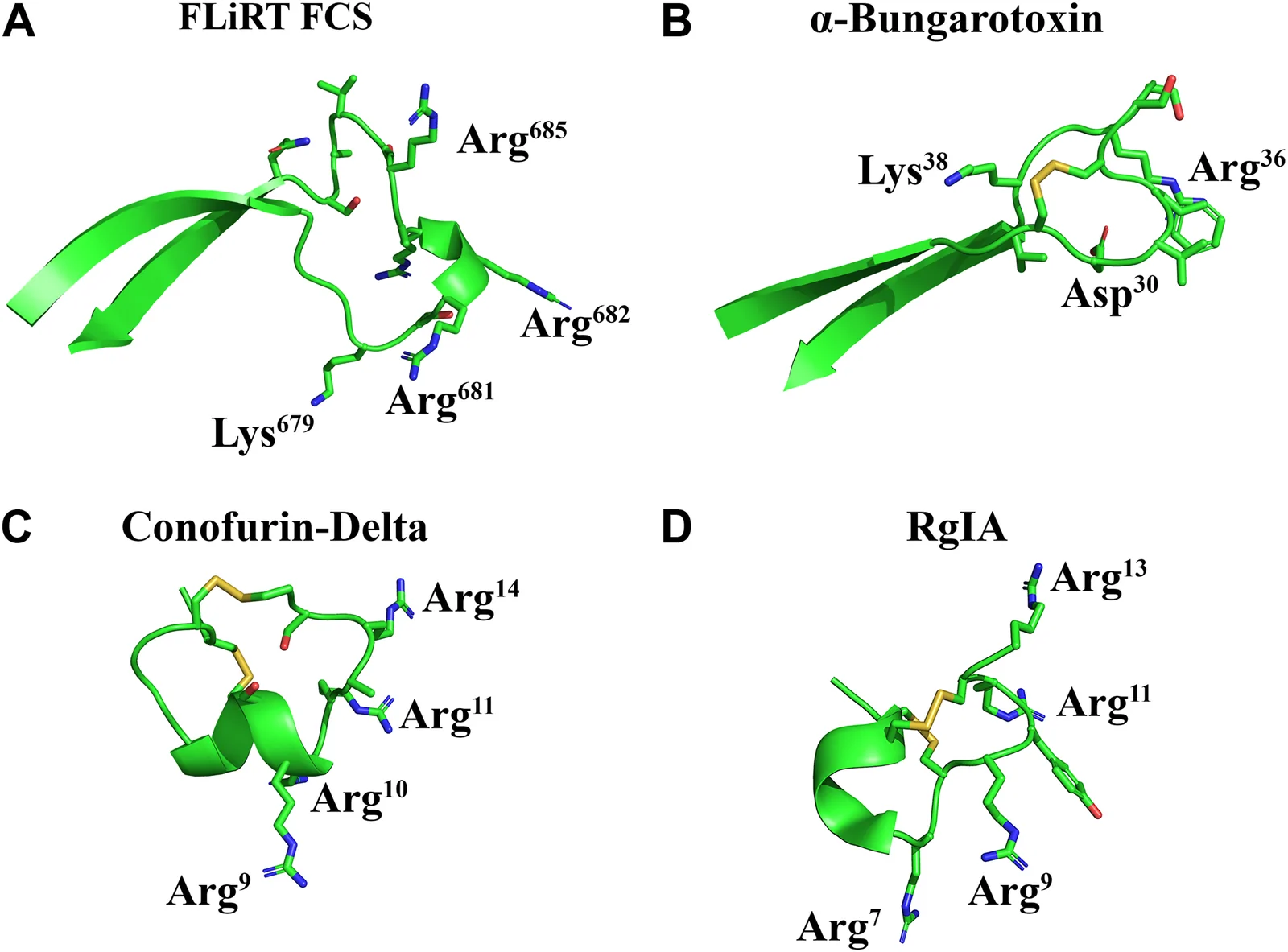
**Peptide ligands that interact with α7 and α9/α10 nAChRs show structural and sequence similarities.** A, cartoon rendering of residues ^670^Ile-Thr^696^ of the FLiRT SARS-CoV-2 spike protein. Residues corresponding to the FLiRT peptide (^679^KSRRRARSVASQ^690^) are shown with the side chains as sticks. *B*, cartoon rendering of residues ^21^Asn-Cys^48^ of α-bungarotoxin (PDB:7KOO); residues ^29^CDVFCSSRGKV^39^ are shown with the side chains as *sticks* and the disulfide bonds between ^21^Cys-Cys^33^ in *yellow* (51). Labeled residues have previously been shown to important for binding to α7 nAChRs (52, 53, 54). *C*, cartoon rendering of the α-conopeptide conofurin-Delta (GCCSHPACSRRRARSC) (PDB:9BAF) (14); residues ^9^SRRRARS^15^ are shown with the side chains as sticks and the disulfide bonds between ^2^Cys-Cys^8^ and ^3^Cys-Cys^16^ in *yellow*. Labeled residues have previously been shown to important for binding to α7 and α9α10 nAChRs (14). *D*, cartoon rendering of α-conopeptide RgIA (GCCSDPRCRYRCR) (PDB: 2JUQ) (34); residues ^7^RCRYRCR^13^ are shown with the side chains as sticks and the disulfide bonds between ^2^Cys-Cys^8^ and ^3^Cys-Cys^12^ in *yellow*. Labeled residues have previously been shown to important for binding to α9α10 nAChRs (34, 55, 56). A previously obtained cyro-EM structure of the WT spike-protein (PDB: 6XR8) (57) was subsequently refined using computational modeling to visualize residues of the FCS that were poorly resolved (58). The refined model was subsequently modified here using PyMOL (59) to depict the FLiRT FCS (*A*).

SARS-CoV-2 expresses a surface glycoprotein that is essential for interaction with the angiotensin-converting enzyme-2 (ACE2) and viral infectivity in humans (1, 33). Unlike closely related viruses, the spike protein of SARS-CoV-2 has a stretch of amino acids that forms a cleavage site for the enzyme furin. It has been proposed that this FCS shares sequence similarities with peptides found in the venom of elapid snakes as well as marine cone snails (Table 1) (14, 17). Furthermore, computational modeling of a spike-protein cryo-EM structure revealed that the FCS and flanking residues (Fig. 8*A*) structurally resemble regions of three-finger neurotoxins that interact with the α7 and α9α10 nAChRs and include α-bungarotoxin (Fig. 8*B*). There are also similarities in the structure of an α-conopeptide where the amino-acid sequence of the Delta FCS (^680^SRRRARS^686^) was inserted in place of the α-conopeptide’s native residues (Fig. 8*C*) (14). The Arg residues of this construct were shown by molecular dynamics simulation to interact with α7, α9, and α10 subunits. We note that native α-conopeptides RgIA (Fig. 8*D*) and ArIB (Table 5) are also rich in Arg residues, and those of RgIA have been shown to be critical for activity on α9α10 nAChRs (34).

There are several notable findings in this study with respect to the nicotinic receptor hypothesis of COVID-19. First, previous studies that assessed the interaction between spike-protein constructs and α7 nAChRs yielded results that might suggest that nAChRs are not involved in the pathology of COVID-19 (25). By contrast, other reports did show a functional interaction between spike-protein constructs and α7 nAChRs (12, 35). Furthermore, computational studies suggest that the spike protein may bind to both α7 and α9α10 subtypes (13, 36). Here we show that not only do peptides corresponding to the SARS-CoV-2 FCS interact with human α7, α9, α10, and α9α10 nAChRs, but they do so in a virus-variant and nAChR subtype dependent manner (Fig. 1, *A*–*D* and Table 2). A striking example can be observed with the WT, Alpha, and Delta peptides activity on the α7 subtype. The WT FCS has a Pro residue at position 681 that mutated to Arg in the Delta variant and to His in the Alpha variant. Of these three peptides, only Delta showed activity on α7 nAChRs further highlighting the importance of Arg residues for activity. Similar results can be observed by comparing the activity of the WT and Delta peptides on α9 homomers. Interestingly, α10 nAChRs were sensitive to inhibition by all the FCS peptides. Lastly, only the FLiRT peptide showed substantial activity on α3β2 nAChRs, suggesting that physiological processes other than those of the immune system might also be affected by SARS-CoV-2 infection (Fig. 5*B*). For example, cardiac, superior cervical, nodose, and stellate ganglion neurons are known to express α3β2 nAChRs and are involved in the control of cardiac function (37). Patients with SARS-CoV-2 infection experience multisystem complications including cardiac impairment, but to our knowledge the role of α3β2 nAChRs in COVID-19 pathophysiology is unknown.

A second important finding of this study concerns the effects of the FCS peptides on the function of human monocytic THP-1 cells. Antagonism of THP-1 nAChRs by the FCS peptides prevented inhibition of BzATP-evoked IL-1β release by ACh (Figs. 2, 6, and S2). Interference of this anti-inflammatory mechanism by the spike protein could, in part, underlie the basis for the cytokine storm observed in severe cases of COVID-19. Selective α-conopeptide antagonists of α7 (ArIB∗) and α9α10 (RgIA4) nAChRs showed similar effects in the IL-1β release assay. Interestingly, ArIB∗ and RgIA4 inhibited the effects of ACh by similar magnitudes, which might suggest two possibilities. The first is that there may be a heteromeric receptor composed of α7 and α10 subunits that is antagonized by both ArIB∗ and RgIA4. α7 and α10 subunits might also combine with other subunits to form a subtype that is sensitive to both α-conopeptides. Second, we do not know how much inhibition of the ACh response is required to completely prevent the effects on BzATP-evoked IL-1β release. We addressed the first possibility by assessing THP-1 cells by qPCR for the expression of other nicotinic subunit RNAs including those for α3, α4, and β2 in addition to α7, α9, and α10 subunits (Fig. 3). The qPCR experiments revealed several notable findings. First, although expression of α4β2 nAChRs has been reported in THP-1 cells (38), we did not detect RNA for α4 subunits. Furthermore, RNA for α9 subunits was absent in all but two samples, suggesting low expression of the CHRNA9 gene under the conditions used here. Similar results have been found in a previous study (39). Interestingly, RNAs for α3 and β2 subunits were detected in all samples, but not all replicates crossed the 40-cycle threshold of detection, also suggesting very low expression levels of these genes. The two most abundant nicotinic RNAs detected were for α7 and α10 subunits, and this coupled with the relative absence of α3, α4, α9, and β2 subunit RNA suggests that under the conditions used in this study, THP-1 cells may predominantly express nAChRs containing α7 and/or α10 subunits.

COVID-19 remains a serious global health concern, particularly given that new variants of SARS-CoV-2 continue to emerge with some variants becoming more infectious than their progenitors (40). New variants often have mutations in the FCS (Table 1) that not only enhance infectivity (40, 41, 42, 43, 44) but also appear to enhance potency on some nAChR subtypes (Fig. 1) (14, 35). There are 16 genes that encode the various human nAChR subunits, which combine to form myriad subtypes with different pharmacological profiles. COVID-19 is a multisystem pathology that affects not only the respiratory system, but also other physiological systems that express nAChRs, particularly the immune system. In this study, we used THP-1 monocytes to demonstrate the effects of nAChR inhibition by FCS peptides on IL-1β release. Similar studies have been conducted using other selective agonists and antagonists of α7 and α9α10 to examine nAChR involvement in modulating the release of TNF-α, IL-6, and IL-10 from several monocyte/macrophage cell lines and from primary cells using human whole blood cultures and mouse bone-marrow derived macrophages (27, 45, 46). Other examples of systems that could be affected by nAChR inhibition by the spike protein might include neurons that modulate cardiovascular system function and express α3β2 nAChRs and those of the gastrointestinal tract that abundantly expresses α3β4 nAChRs (47). Furthermore, SARS-CoV-2 infection has been reported to induce a myasthenia gravis-like syndrome (48) which may be partially due to inhibition of the α-bungarotoxin sensitive nAChR found at the neuromuscular junction. Lastly, extended cognitive impairment, a feature of Long-COVID, has been observed in a subpopulation of COVID-19 patients, and spike protein fragments have been found in cerebral meninges and the brain long after resolution of the initial viral infection (49). Inhibition of brain α7 and α4β2 nAChRs by the spike protein might be involved in the cognitive impairment associated with Long-COVID. These disparate clinical features-neurological, autonomic, gastrointestinal, neuromuscular, and cognitive-could all be exacerbated by a common mechanism involving spike-protein-mediated perturbation of nAChR function. Establishing the extent and physiological relevance of this interaction will be critical for informing diagnostic strategies and guiding the development of targeted therapeutics.

## Experimental procedures

### Materials

Acetylcholine chloride was obtained from Tocris. Sodium chloride, potassium chloride, calcium chloride dihydrate, magnesium chloride hexahydrate, and 4-(2-hydroxyethyl)-1-piperazineethanesulfonic acid were obtained from Sigma-Aldrich.

### Peptide synthesis

Peptides were synthesized using the automated microwave synthesizer Liberty Blue 2.0 equipped in HT12 from CEM Corporation at 50 μmol scale following Fmoc protocols. Peptides were assembled on a preloaded Fmoc-Rink Amide MBHA resin (substitution: 0.41 mmol/g) from NovaBiochem (now part of the MilliporeSigma). Standard amino acids were purchased from AAPPTec, LLC with the side-chain protection as follows: Ser, tert-butyl (tBu); Asn and Gln, trityl (Trt); Lys and His, tert-butyloxycarbonyl (Boc); and Arg, 2,2,4,6,7-pentamethyldihydrobenzofuran-5-sulfonyl (Pbf). Fmoc deprotection was performed for 1 min with 10% (v/v) piperidine (Thermo Scientific; Waltham, MA, USA) in dimethylformamide (DMF; Fisher Scientific) at 90^o^C. A five-fold excess of amino acid (0.2 M solution in DMF) was used for each coupling reaction. Coupling activation was achieved with 1 equivalent of 0.5 M OxymaPure (CEM Corporation) and 2 equivalents of 0.5 M N,N′-diisopropylcarbodiimide (Advanced ChemTech) in DMF at 90 °C for 2 min. Following final Fmoc deprotection the N-terminus of the peptides was acetylated with 10% acetic anhydride (Fisher Scientific) in DMF at 65^o^C for 2 min. After the synthesis, the resin was washed with dichloromethane and dried under vacuum.

Peptides were removed from the resin at 40 °C using Razor cleavage system (CEM Corporation) and 3 ml of the following cleavage cocktail: trifluoroacetic acid (TFA, Fisher Scientific)/triisopropylsilane (MilliporeSigma)/2,2′-(ethylenedioxy)diethanethiol/water 92.5/2.5/2.5/2.5 (%/%). After 40 min the cleavage mixture was filtered and precipitated with 10 ml of ice-cold methyl-tert-butyl ether (MTBE; Fisher Scientific). The crude peptide was then precipitated by centrifugation at 7000×*g* for 7 min and washed twice with 10 ml of ice-cold MTBE. The crude peptides were dissolved in 0.1% TFA in water and purified by reverse-phase high performance liquid chromatography (RP-HPLC) using a preparative C18 column using a buffer gradient of 5 to 45% B60 (60% vol/vol acetonitrile (Fisher Scientific), 40% vol/vol water, and 0.092% vol/vol TFA) at a rate of 1% per minute and a flow rate of 20 ml/min. The masses of the peptides were verified by electrospray mass spectrometry (Table S1) at the University of Utah Department of Chemistry (Salt Lake City, Utah, USA). The purity of the peptides was determined by RP-HPLC using an analytical C18 column and the same gradient used for purification but with a flow rate of 1 ml/min. All peptides had purity levels ≥95%.

### Electrophysiology

Protocols (No. 17–07020) for obtaining oocytes from *X. laevis* frogs were approved by the University of Utah Institutional Animal Care and Use Committee. Detailed methods for conducting TEVC experiments of human nAChRs heterologously expressed in *X. laevis* oocytes have been previously described (14). Briefly, the oocytes were continuously perfused by gravity at a rate of ∼3 ml/min with frog saline composed of 96 mM NaCl, 2.5 mM KCl, 1.8 mM CaCl_2_, 1.0 mM MgCl_2_, 1 mg/ml bovine serum albumin buffered to pH 7.4 with (4-(2-hydroxyethyl)-1-piperazineethanesulfonic acid. The perfusion system consisted of a series of solenoid valves (NResearch Inc) controlled by LabVIEW (RRID:SCR_014325) software (National Instruments) operating on a personal computer. To assess peptide activity, the oocyte membranes were clamped at a holding potential of −70 mV using a Warner Instruments Oocyte Clamp OC-725C (Warner Instruments) and stimulated with 100 μM acetylcholine (ACh) until stable baseline responses were observed. The ACh-evoked currents were acquired and sampled at 50 Hz using a USB-6009 digital acquisition system (National Instruments) and filtered at 5 Hz using an F1B1 low pass Bessel filter (Frequency Devices). Oocytes expressing the α7 subtype were pulsed with ACh for 500 ms, to minimize open-channel block by ACh, and 1000 ms for all other subtypes. Once stable responses were observed, saline solution containing FCS peptide was perfusion applied for concentrations ≤3 μM. For concentrations ≥10 μM, the peptides were applied in a static bath for 5 min. The ACh-evoked current amplitudes in the presence of the peptides were compared to currents in response to control applications of saline. All data were analyzed using Prism 10.5.0 (RRID:SCR_002798) (GraphPad). The IC_50_ values are provided with the corresponding 95% CI. The error bars indicate ± the SD of ≥4 individual determinations. For qualitative comparison of peptide activity among the different peptide/receptor combinations, potency was defined as low if the IC_50_ values were >10 μM.

#### Cell culture

THP-1 cells (RRID:CVCL_0006) were purchased from the German Collection of Microorganisms and Cell Cultures, Cat# ACC16) and cultured in RPMI-1640 (Capricorn, Ebsdorfergrund, Germany or Thermo Fisher) medium supplemented with 10% (v/v) fetal bovine serum (FBS; Capricorn, Ebsdorfergrund, Germany, Cat# FBS-16A or Thermo Fisher), 50 μg/ml penicillin and 50 units/ml streptomycin, and maintained in an incubator at 37 °C in a humidified atmosphere of 5% CO_2_/95% air. U87-MG (RRID: CVCL_0022) cells were obtained from ATCC and cultured under similar conditions as those for THP-1 cells except that DMEM (Thermo Fisher) was used as the culture medium supplemented with the above listed serum and antibiotics.

### Quantitative real-time PCR

Total RNA was extracted from THP-1 and U87-MG cells from independent cultures using a Qiagen RNAesy Mini Kit (Qiagen) and subsequently treated on column with DNAse I (Qiagen) to degrade genomic DNA. The purity of the RNA was assessed by measuring the A_260_/A_280_ ratio using a BioTek Epoch spectrophotometer (Agilent Technologies). cDNA was reverse transcribed from RNA (2 μg/20 μl) reaction using High-Capacity cDNA Reverse Transcription Kit (Applied Biosystems) following the manufacturer’s instructions. The cycling conditions for the reverse-transcription PCR (RT-PCR) were as follows: step 1, 25 °C for 10 min; step 2, 37 °C for 120 min; and step 3, 85 °C for 5 min and were achieved using a C1000 Touch Thermocycler (Bio-Rad Laboratories Inc). The presence of residual genomic DNA was further assessed by omitting reverse transcriptase in the RT-PCR reactions and subsequently used in quantitative real-time PCR (qPCR) reactions.

Quantitative real-time PCR was performed to assess the expression of mRNA transcripts in THP-1 and U87-MG cells using inventoried TaqMan Gene Expression Assays (Thermo Fisher, Waltham MA, USA) according to manufacturer’s instructions. Detailed information about the individual TaqMan probes can be found in Table S2. Each 20 μl qPCR reaction was performed in triplicate and assembled using 10 μl of TaqMan 2X Fast Advanced Master Mix (containing AmpliTaq Fast DNA Polymerase, uracil-N glycosylase (UNG), dNTPs with dUTP, and ROX dye (passive reference)), 1 μl of a 20X hydrolysis probe set, and nuclease-free water containing 50 ng of cDNA (derived from RNA concentrations). Samples were prepared in 96-well plates (BioRad) and the gene targets were amplified using a Bio-Rad CFX96 Touch Real-Time PCR Detection System. The qPCR thermal cycling program used was as follows: 1 cycle of 50° C for 2 min followed by 95 °C for 20 s (to activate, and then inactivate UNG which degrades any carry-over DNA), 40 cycles of 1 s denaturation at 95 °C followed by 20 s of annealing and extension at 60 °C (50).

### Cytokine release assay

THP-1 cells were cultured using conditions similar to those used for qPCR experiments. In brief, THP-1 cells were cultured in RPMI 1640 medium (Capricorn, Ebsdorfergrund, Germany) supplemented with 10% vol/vol FBS from Capricorn and maintained in an atmosphere of 95%/5% air/CO_2_ at 37 °C. On the day of the experiment, 0.5 × 10^6^ cells were seeded in 48-well plates in a total volume of 500 μl/well of FBS-free RPMI medium. Cells were primed with 1 μg/ml LPS (*E. coli* O26:B6, Merck, Darmstadt, Germany, Cat# L2654) for 5 h, followed by 40 min of 2′(3′)-O-(4-benzoylbenzoyl)adenosine-5′-triphosphate (BzATP) tri(triethylammonium) salt; Jena Bioscience, Jena, Germany) treatment with or without test compounds. The supernatants were collected and stored for later IL-1β measurements using the Human IL-1β/IL-1F2 DuoSet ELISA from R&D Systems. Cell viability was estimated by measuring lactate dehydrogenase (LDH) activity in cell-free supernatants using the CytoTox 96 Non-Radioactive Cytotoxicity Assay (Promega, Madison WI, USA). LDH activities were expressed as a percentage of total LDH activity from lysed control cells. Data for LDH activity were analyzed using SPSS (RRID:SCR_002865) (Version 29.0.2.0, IBM, Armonk, NY, United States) and visualized using Inkscape (RRID:SCR_014479) version 0.48.5 r10040 (Free and Open Source Software licensed under the GPL). See Table S3 for a summary of the LDH assay results.

### Statistical analysis

#### Two-electrode voltage-clamp electrophysiology

Prior to analysis, all data were evaluated for normality using a Shapiro-Wilks test to determine the appropriate test for statistical differences. The TEVC electrophysiology data used to determine peptide potency were analyzed by non-linear regression using GraphPad Prism (RRID:SCR_002798) (GraphPad Inc) statistical analysis software and expressed as the estimated IC_50_ value and the corresponding 95% confidence interval. The error bars indicate the mean ± SD and are provided to evaluate the variance of the data. For TEVC electrophysiology data sets where a single concentration of ArIB∗, RgIA, or PeIA∗ were used, the data were compared to a theoretical response value of 100% (*i.e.*, no effect) using an unpaired *t* test and shown with error bars denoting mean ± SD.

#### Cytokine release assay

Release of IL-1β from THP-1 monocytes was determined using ELISA. The effects of FCS and α-conopeptides on the inhibition of BzATP release by ACh were determined using a Friedman test and the linear two-stage set-up method of Benjamani, Krieger, and Yekutieli (BKY) to control for false discovery rates. Results were considered statistically significant if *p* < 0.05 and a false discovery if q > 0.05. The error bars indicate the mean ± SD.

### Quantitative real-time PCR

Quantitative real-time PCR data were collected in triplicate for each sample/gene combination, and the C_q_ value ± SEM was determined. The C_q_ values for the nicotinic subunit genes were normalized to the geometric mean of ACTB and GAPDH C_q_ values using the log_10_(2^-ΔCq^) method where ΔCq = (mean nAChR gene Cq minus the geometric mean of the reference gene Cq value) and are provided for transparency. RNAs with C_q_ threshold values ≥ 40 were considered below level of detection. The error bars indicate the mean ± SD.

## Data availability

Data for this work are available upon reasonable request to Arik J. Hone, uuneurotox@yahoo.com.

## Supporting information

This article contains supporting information.

## Conflict of interest

The authors declare that they have no conflicts of interest with the contents of this article.
