# Supplementary material for: Peptides containing SARS-CoV-2 spike-protein residues are antagonists of α7 and α9α10 nAChRs and modulate interleukin-1β release from human monocytic THP-1 cells
Source: J Biol Chem. 2026 Aug 5;302(10):113391. doi: 10.1016/j.jbc.2026.113391 (PMC13579935; doi:10.1016/j.jbc.2026.113391)
Supplement: Supplementary file 1 [file mmc1.docx]

Peptides containing SARS-CoV-2 spike-protein residues are antagonists of α7 and α9α10 nAChRs and modulate interleukin-1β release from human monocytic THP-1 cells

Arik J. Hone^1,2^, Katrin Richter^3,4^, Zoha Riaz^1^, Joseph Wakkuri^1^, Bassel Tekarli^1^, Joanna Gajewiak^1^, Veronika Grau^3^, and J. Michael McIntosh^1,5,6^

^1^School of Biological Sciences, University of Utah, Salt Lake City, Utah, 84112, USA; ^2^MIRECC, George E. Wahlen Veterans Affairs Medical Center, Salt Lake City, Utah, 84148, USA; ^3^Laboratory of Experimental Surgery, Department of General and Thoracic Surgery, German Center for Lung Research (DZL), Cardiopulmonary Institute (CPI), Justus-Liebig University Giessen, Giessen, 35392, Germany; ^4^Department of Natural Sciences, Bonn-Rhein-Sieg University of Applied Sciences, Rheinbach, 53359, Germany; ^5^Department of Psychiatry, University of Utah, Salt Lake City, Utah, 84112, USA; ^6^George E. Wahlen Veterans Affairs Medical Center, Salt Lake City, Utah, 84148, USA.

**Keywords:** SARS-CoV-2, COVID-19, spike protein, furin cleavage-site, cytokine storm, nicotinic acetylcholine receptors, α-conopeptides

**Running title:** SARS-CoV-2 spike-protein inhibits nAChRs

To whom correspondence should be addressed:

Arik J. Hone Ph.D.

School of Biological Sciences, University of Utah

Salt Lake City, Utah, 84112, USA

uuneurotox@yahoo.com

Tel. (801) 581-8370

J. Michael McIntosh M.D.

School of Biological Sciences, University of Utah

Salt Lake City, UT, USA, 84112

mcintosh.mike@gmail.com

Tel. (801) 581-8370

**Figure S1.**

**Peptides containing SARS-CoV-2 spike-protein residues have no direct effects on LPS-evoked IL-1β release from monocytic THP-1 cells**. THP-1 monocytes were primed with LPS (1 µg/ml, 5 hours), and the release of IL-1β was quantified by ELISA as described in Experimental procedures. Potential direct effects of the FCS peptides on LPS-evoked IL-1β release were assessed by incubating LPS-primed cells with indicated FCS peptide (10 µM) in the absence of BzATP or ACh for 40 min; no effects on the amount of IL-1β evoked by LPS were observed. Similarly, no effects were observed by exposing the cells to α-conopeptides ArIB* (500 nM) or RgIA4 (200 nM). The error bars in A and B represent the SD of the data obtained from 6 individual biological replicates. The data were analyzed for significance using a Friedman test followed by a BKY post hoc test. Results were considered a discovery if q ≤ 0.05; see Table 4 for a summary of the results. ArIB*, [V11L,V16D]ArIB.

**Figure S2.**

**Peptides containing SARS-CoV-2 spike-protein residues prevent ACh-mediated inhibition of BzATP-evoked IL-1β release from monocytic THP-1 cells**. THP-1 monocytes were primed with LPS (1 µg/ml, 5 hours) then stimulated with BzATP (100 µM; 40 min) to evoke the release of IL-1β that was quantified by ELISA as described in Experimental procedures. (A) The ability of the FCS peptides to prevent inhibition of IL-1β release by ACh (100 µM) was assessed by incubating the cells with ACh and the indicated FCS peptide (3 µM) in the presence of BzATP. The amount of BzATP-evoked IL-1β released in the presence of ACh was normalized to the amount evoked by BzATP in the absence of ACh. Acetylcholine significantly inhibited IL-1β release under these conditions. Peptide effect was assessed for significance by comparing the amount of IL-1β obtained in the presence of the peptide to that obtained in the BzATP + ACh group. All peptides but WT could prevent the inhibitory effect of ACh on the BzATP-induced IL-1β release at the concentration tested. (B) BzATP-evoked IL-1β release in the absence of ACh and in the presence of FCS peptides. No inhibition of IL-1β release was found in the absence of ACh. The error bars in A and B represent the SD of the data obtained from six independent experiments. The data were analyzed for significance using a Friedman test followed by a BKY post hoc test. Results were considered a discovery if q ≤ 0.05; see Table 5 for a summary of the results. ArIB*, [V11L,V16D]ArIB.

**Figure S3.**

**The FLiRT SARS-CoV-2 spike-protein peptide and α-conopeptides have no direct effect on LPS-evoked IL-1β release from monocytic THP-1 cells**. THP-1 monocytes were primed with LPS (1 µg/ml, 5 hours). Thereafter, the cells were exposed to FLiRT or α-conopeptides for 40 min, and the release of IL-1β quantified by ELISA as described in Experimental procedures. Potential direct effects of the FLiRT peptide on LPS-evoked IL-1β release were assessed by incubating the cells with the peptide at the indicated concentrations. No effects on the amount of IL-1β evoked by LPS were observed as a result of exposure to the peptide. Similarly, no effects were observed by exposing the cells to α-conopeptides ArIB* (100 nM), RgIA4 (200 nM), or PeIA* (10 nM). The error bars in A and B represent the SD of the data obtained from 6 independent experiments. The data were analyzed for significance using a Friedman test followed by a BKY post hoc test. Results were considered a discovery if q ≤ 0.05; see Table 6 for a summary of the results. ArIB*, [V11L,V16D]ArIB; PeIA*, [S9H,V10A,E14N]PeIA.

Figure S4.

**α-Conopeptide OmIA inhibits human α7 but not** **α9α10 nAChRs.** *X. laevis* oocytes were used to assess the ability of OmIA to inhibit ACh-evoked currents mediated by the indicated nAChR subtypes as described in Experimental procedures. Concentration-response analysis showing that OmIA inhibited α7 nAChRs with an IC_50_ value of 238 (207-273) nM; values in parentheses indicate the 95% CI. By contrast, the ACh responses mediated by α9α10 nAChRs were 85 ± 8% of control values in the presence of OmIA (10 µM). The error bars represent the SD of the data from 5 individual oocytes.

| Table S1. Mass spectrometry of synthetic peptides | | |
| --- | --- | --- |
| **peptide** | **calculated M^+^ (Da)** | **observed M^+^ (Da)** |
| WT | 1369.70 | 1369.74 |
| Alpha | 1409.75 | 1409.76 |
| Delta | 1428.80 | 1428.80 |
| Omicron | 1423.80 | 1423.80 |
| FLiRT | 1442.84 | 1441.83 |
| All masses were determined by electrospray mass spectrometry. | | |

| **Table S2. TaqMan gene expression assays** | | | |
| --- | --- | --- | --- |
| **assay ID** | **context sequence** | **gene** | **accession number** |
| Hs01088199_m1 | ACCTGTGGCTCAAGCAAATCTGGAA | *CHRNA3* | NM_001166694.1 |
| Hs00181247_m1 | ACACAGACTTCTCGGTGAAGGAGGA | *CHRNA4* | NM_001256573.1 |
| Hs01063373_m1 | CTCTATAACAGTGCTGATGAGCGCT | *CHRNA7* | NM_001190455.2 |
| Hs00395558_m1 | TGCCCCTGATAGGTAAATACTACAT | *CHRNA9* | NM_017581.3 |
| Hs00220710_m1 | TAACAAAGCCGACGCGCAGCCTCCA | *CHRNA10* | NM_020402.2 |
| Hs00181267_m1 | ACAACAATGCTGACGGCATGTACGA | *CHRNB2* | NM_000748.2 |
| Hs00609520_m1 | GACTCATGACCACAGTCCATGCCAT | *GAPDH* | NM_001256799.1 |
| Hs01060665_g1 | CCCAGGCACCAGGGCGTGATGGTGG | *ACTB* | NM_001101.3 |

| **Table S3. Monocytic THP-1 cell death under the conditions used in this study as measured by the lactate dehydrogenase (LDH) assay** | | |
| --- | --- | --- |
|  | **cell death (%)** | **n** |
| **experimental condition** |  |  |
| LPS (1 µg/ml) | 6 ± 2 | 24 |
| BzATP | 5 ± 2 | 21 |
| BzATP + ACh | 5 ± 2 | 12 |
| BzATP + ACh + WT (3 µM) | 4 ± 1 | 6 |
| BzATP + WT (3 µM) | 3 ± 1 | 3 |
| BzATP + ACh + WT (10 µM) | 4 ± 1 | 6 |
| BzATP + WT (10 µM) | 4 ± 1 | 6 |
| BzATP + ACh + Alpha (3 µM) | 3 ± 1 | 6 |
| BzATP + Alpha (3 µM) | 4 ± 1 | 3 |
| BzATP + ACh + Alpha (10 µM) | 4 ± 1 | 6 |
| BzATP + Alpha (10 µM) | 4 ± 1 | 6 |
| Alpha (10 µM) | 4 ± 1 | 3 |
| BzATP + ACh + Delta (3 µM) | 4 ± 1 | 6 |
| BzATP + Delta (3 µM) | 3 ± 0 | 3 |
| BzATP + ACh + Delta (10 µM) | 4 ± 1 | 6 |
| BzATP + Delta (10 µM) | 4 ± 1 | 6 |
| Delta (10 µM) | 4 ± 1 | 3 |
| BzATP + ACh + Omicron (3 µM) | 4 ± 1 | 6 |
| BzATP + Omicron (3 µM) | 3 ± 1 | 3 |
| BzATP + ACh + Omicron (10 µM) | 3 ± 1 | 6 |
| BzATP + Omicron (10 µM) | 4 ± 1 | 6 |
| Omicron (10 µM) | 4 ± 1 | 3 |
| BzATP + ACh + FLiRT (3 µM) | 4 ± 2 | 12 |
| BzATP + FLiRT (3 µM) | 4 ± 2 | 9 |
| FLiRT (3 µM) | 8 ± 3 | 9 |
| BzATP + ACh + FLiRT (10 µM) | 4 ± 2 | 12 |
| BzATP + FLiRT (10 µM) | 4 ± 1 | 12 |
| FLiRT (10 µM) | 7 ± 3 | 9 |
| BzATP + ACh + ArIB* (100 nM) | 5 ± 2 | 6 |
| BzATP + ArIB* (100 nM) | 5 ± 1 | 6 |
| ArIB* (100 nM) | 8 ± 3 | 9 |
| BzATP + ACh + ArIB* (500 nM) | 5 ± 2 | 15 |
| BzATP + ArIB* (500 nM) | 4 ± 2 | 15 |
| ArIB* (500 nM) | 4 ± 1 | 3 |
| BzATP + ACh + RgIA4 (200 nM) | 5 ± 2 | 21 |
| BzATP + RgIA4 (200 nM) | 5 ± 2 | 21 |
| RgIA4 (200 nM) | 7 ± 3 | 9 |
| BzATP + ACh + PeIA* (10 nM) | 6 ± 3 | 6 |
| BzATP + PeIA* (10 nM) | 5 ± 2 | 6 |
| PeIA* (10 nM) | 8 ± 3 | 6 |
| BzATP + ACh + OmIA (10 µM) | 5 ± 2 | 9 |
| BzATP + OmIA (10 µM) | 5 ± 2 | 9 |
| OmIA (10 µM) | 5 ± 2 | 9 |
| All experiments were performed in the presence of LPS (1 µg/ml). The ‘±’ represents the SD of the data obtained from (n) individual biological replicates; ‘nd’, indicates values not determined; ArIB*, [V11L,V16D]; PeIA*, [S9H,V10A,E14N]PeIA. | | |

| **Table S4. LPS evoked IL-1β release from monocytic THP-1 cells** | |
| --- | --- |
|  | **IL-1β release (pg/ml)** |
| **experimental condition** |  |
| LPS (1 µg/ml) + WT (10 µM) | 5 ± 2 |
| LPS (1 µg/ml) + Alpha (10 µM) | 5 ± 2 |
| LPS (1 µg/ml) + Delta (10 µM) | 5 ± 2 |
| LPS (1 µg/ml) + Omicron (10 µM) | 5 ± 2 |
| LPS (1 µg/ml) + FLiRT (10 µM) | 6 ± 2 |
| LPS (1 µg/ml) + ArIB*(500 nM) | 6 ± 2 |
| LPS (1 µg/ml) + RgIA4 (200 nM) | 6 ± 2 |
| The ‘±’ represents the SD of the data obtained from 6 individual biological replicates; ArIB*, [V11L,V16D]ArIB. | |

| **Table S5. BzATP-evoked IL-1β release from monocytic THP-1 cells** | | |
| --- | --- | --- |
|  | **normalized IL-1β release (%)** | |
|  | **ACh + BzATP** | **BzATP - ACh** |
| **experimental condition** |  |  |
| BzATP | 100 ± 57* | 100 ± 52* |
| BzATP + ACh | 12 ± 6* | - |
| WT (3 µM) | 60 ± 39 | 85 ± 21 |
| Alpha (3 µM) | 79 ± 42 | 87 ± 35 |
| Delta (3 µM) | 81 ± 32 | 86 ± 17 |
| Omicron (3 µM) | 111 ± 48 | 83 ± 26 |
| FLiRT (3 µM) | 163 ± 67 | 96 ± 32 |
| All experiments were performed in the presence of LPS (1 µg/ml). The ‘±’ represents the SD of the data obtained from 6 individual biological replicates; the asterisks denote values obtained in parallel experiments presented in Fig. 2 and Table 3 of the main text. The BzATP + ACh column corresponds to Fig. S2A and the BzATP – ACh column to Fig. S2B. | | |

| **Table S6. LPS-evoked IL-1β release from monocytic THP-1 cells** | |
| --- | --- |
|  | **IL-1β release (pg/ml)** |
| **experimental condition** |  |
| LPS (1 µg/ml) | 4 ± 2 |
| LPS (1 µg/ml) + FLiRT (3 µM) | 3 ± 1 |
| LPS (1 µg/ml) + FLiRT (10 µM) | 3 ± 1 |
| LPS (1 µg/ml) + ArIB* (100 nM) | 4 ± 1 |
| LPS (1 µg/ml) + RgIA4 (200 nM) | 4 ± 2 |
| LPS (1 µg/ml) + PeIA* (10 nM) | 3 ± 1 |
| The ‘±’ represents the SD of the data obtained from 6 individual biological replicates; ArIB*, [V11L,V16D]ArIB; PeIA*, [S9H,V10A,E14N]PeIA. | |
